# Draft Genome Sequence and Annotation of the Obligate Bacterial Endosymbiont *Caedibacter taeniospiralis*, Causative Agent of the Killer Phenotype in *Paramecium tetraurelia*

**DOI:** 10.1128/genomeA.01418-17

**Published:** 2018-01-18

**Authors:** Nestor Zaburannyi, Katrin Grosser, Gilles Gasparoni, Rolf Müller, Martina Schrallhammer, Martin Simon

**Affiliations:** a Molecular Cell Dynamics, Centre for Human and Molecular Biology, Saarland University, Saarbrücken, Germany; b Microbiology, Institute of Biology II, Albert Ludwig University of Freiburg, Freiburg, Germany; c Genetics, Centre for Human and Molecular Biology, Saarland University, Saarbrücken, Germany; d Department of Microbial Natural Products, Helmholtz Centre for Infection Research and Department of Pharmacy, Helmholtz Institute for Pharmaceutical Research Saarland (HIPS), Saarland University, Saarbrücken, Germany; e German Centre for Infection Research (DZIF), Partner Site Hannover-Braunschweig, Hannover, Germany

## Abstract

*Caedibacter taeniospiralis* is an obligate endosymbiont living in the cytoplasm of *Paramecium tetraurelia*. *C. taeniospiralis* causes the so-called killer trait, eliminating intraspecific competitors of its host when released into the medium by the concerted action of the unusual protein structure R-body (refractile body) in addition to an as-yet-unknown toxin.

## GENOME ANNOUNCEMENT

*Caedibacter taeniospiralis* (*Thiotrichales*, *Gammaproteobacteria*) lives in the cytoplasm of its host, *Paramecium tetraurelia* (*Ciliophora*). This unicellular, relatively large (150-µm) eukaryote separates vegetative and sexual functions, possesses germline and somatic nuclei, and is a long-established model in genetics and epigenetics research (1). A special feature of the *Paramecium-Caedibacter* symbiosis is the “killer trait.” A proportion of the endosymbiont population produces an unusual protein structure, the R-body (refractile body) (2). This structure, most likely acting as a delivery device for an unidentified toxin, is responsible for killing symbiont-free, sensitive paramecia after the ingestion of bacteria released from infected strains (3). *Caedibacter-*harboring cells are protected from the lethal effect of their symbionts. Cultivation of *C. taeniospiralis* outside its host has not been accomplished so far.

Total DNA was isolated from exponentially growing paramecia. Their infection status was verified by fluorescence *in situ* hybridization, which enabled detection of intracellular *C. taeniospiralis* 51^T^, as described elsewhere (4). To minimize contamination from food bacteria, *P. tetraurelia* cultures (strain 51K = CCAP 1660/3F) were fed with β-lactam-hypersensitive *Escherichia coli* ΔtolC (5) and treated with 10 µg ml^−1^ ampicillin before DNA isolation (6). Library preparation for whole-genome sequencing used the tagmentation procedure (7). We generated a library of ~500 to 800 bp for subsequent sequencing on an Illumina MiSeq instrument (2 × 300 nucleotides [nt]). After assembly using the ABySS-pe program (8), 24 contigs were assigned to the genome of *C. taeniospiralis*. These can be differentiated from the host genome by their nearly identical coverage and by their GC content of 41.5%, diverging to the host genome’s GC content of 28% (9). Draft genome sequences had a total sequence length of 1.3 Mb (*N*_50_, 55.531 bp), including the previously known plasmid, pKAP, of 41.7 kb (10). The genome contains three rRNA clusters in 16S-23S-5S configurations.

Gene annotation of the draft sequence was carried out using Prodigal (11), tRNAscan-SE (12), and RNAmmer (13) software tools. Next to 36 tRNA genes, 1,080 protein coding sequences could be identified, of which, 787 could be functionally annotated by a similarity search against the eggNOG database with the eggNOG-mapper software (14).

The genome sequence of *C. taeniospiralis* strain 51K will improve our understanding of this organism and its symbiotic interaction with *P. tetraurelia*, including and in addition to the killer trait. The availability of this sequence information enables phylogenomic analyses of the genus *Caedibacter* and will provide a valuable resource for the identification and analysis of toxin candidates, which might have unusual biological properties that explain the need for the R-body as a delivery device.

### Accession number(s).

This whole-genome shotgun project has been deposited at DDBJ/ENA/GenBank under the accession number PGGB00000000. The version described in this paper is version PGGB01000000.
